# Supplementary material for: A Global Screen for Assembly State Changes of the Mitotic Proteome by SEC-SWATH-MS
Source: Cell Syst. 2020 Feb 26;10(2):133–155.e6. doi: 10.1016/j.cels.2020.01.001 (PMC7042714; doi:10.1016/j.cels.2020.01.001)

P49593 | PPM1F_HUMAN | PPM1F KIAA0015 POPX2
Monomer MW [kDa]: 49.831 Monomer expected elution fraction: 46

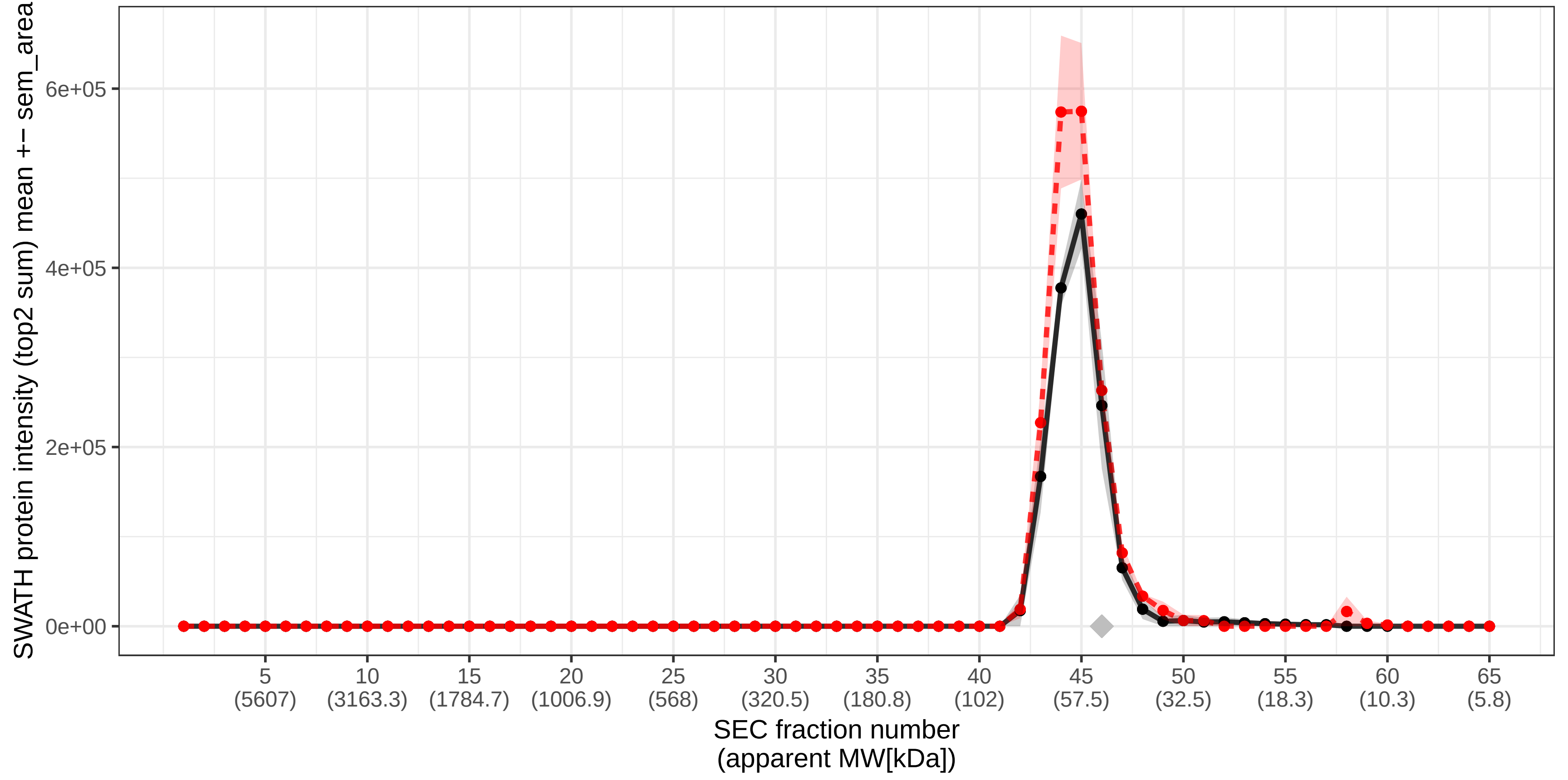

Supplement: Data S1. SEC-SWATH-MS Protein Chromatograms, Related to Figure 1 [file mmc6.zip › SECchrom_P49593_PPM1F_HUMAN_PPM1F_KIAA0015_POPX2.pdf]
